# Supplementary material for: Collagen breaks at weak sacrificial bonds taming its mechanoradicals
Source: Nat Commun. 2023 Apr 12;14:2075. doi: 10.1038/s41467-023-37726-z (PMC10097693; doi:10.1038/s41467-023-37726-z)
Supplement: Supplementary file 1 — Supplementary Information [file 41467_2023_37726_MOESM1_ESM.pdf]

Supplementary Information: Collagen breaks at weak sacrificial bonds taming its mechanoradicals

Benedikt Rennekamp[1,2,3], Christoph Karfusehr[1,3,4], Markus Kurth[1,2], Aysecan Ünal[1,2,3], Debora Monego[1], Kai Riedmiller[1], Ganna Gryn'ova[1,2], David M. Hudson[5], and Frauke Gräter[1,2,3,*]

[1]Heidelberg Institute for Theoretical Studies, Schloss-Wolfsbrunnenweg 35, 69118 Heidelberg, Germany
[2]Interdisciplinary Center for Scientific Computing, Heidelberg University, INF 205, 69120 Heidelberg, Germany
[3]Max Planck School Matter to Life, Jahnstrasse 29, 69120 Heidelberg, Germany
[4]Physics Department and ZNN, Technical University Munich, Coulombwall 4a, 85748 Garching, Germany
[5]Department of Orthopaedics and Sports Medicine, University of Washington, Seattle, WA, 98195, USA
[*]frauke.graeter@h-its.org

March 25, 2023

1 Supplementary Figures and Tables

1.1 Data for the PYD crosslink

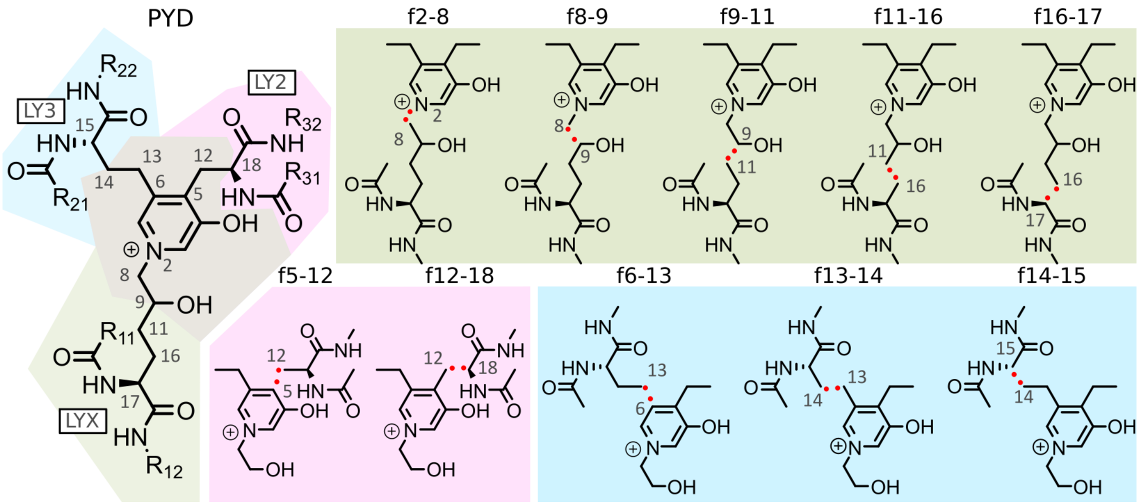

Suppl. Fig. 1: **Internal nomenclature of molecular structures used for BDE calculations of the PYD crosslink.** The presented nomenclature corresponds to the following Suppl. Tables 1-2. The "a" fragment always includes the central nitrogen, the "b" fragment always the truncated backbone.

Molecule	ZPVE [Ha]	H0K [Ha]	H298K [Ha]
0_PYD_b	-1167.81640	-1167.34701	-1167.31685
1_PYD_l	-1089.26818	-1088.85407	-1088.82669
2_PYD_r	-1049.99417	-1049.60825	-1049.58210
f11-16b	-494.71218	-494.54312	-494.52969
f11-16a	-672.95343	-672.66416	-672.64603
f14-15a	-633.67634	-633.41467	-633.39786
f5-12a	-555.09102	-554.88329	-554.86945
f13-14a	-594.41633	-594.18073	-594.16598
f12-18a	-594.41509	-594.17963	-594.16479
f12-18a depr	-594.02805	-593.80541	-593.79083
f2-8b	-687.69188	-687.43480	-687.41585
f2-8a	-479.94870	-479.74752	-479.73474
f6-13a	-555.10169	-554.89306	-554.87969
f16-17b	-455.46470	-455.32179	-455.30981
f16-17a	-712.22699	-711.90990	-711.89039
f9-11a	-633.68808	-633.42542	-633.40879
f9-11b	-533.98481	-533.78833	-533.77329
f8-9b	-648.42901	-648.19758	-648.18042
f8-9a	-519.23885	-519.00942	-518.99564
f8-9a-H-capped	-519.90996	-519.66678	-519.65253
f8-9b-H-capped	-649.09141	-648.84678	-648.82959
f12-18-depr-a-H-capped	-594.66872	-594.43435	-594.41912
f16-17a-H-capped	-712.89938	-712.56813	-712.54870
f16-17b-H-capped	-456.11102	-455.95560	-455.94338
f11-16b-H-capped	-495.38826	-495.20462	-495.19139
f11-16a-H-capped	-673.62836	-673.32479	-673.30686
f6-13a-H-capped	-555.80212	-555.58054	-555.56716
f14-15a-H-capped	-634.35094	-634.07416	-634.05775
f9-11a-H-capped	-634.35030	-634.07402	-634.05750
f9-11b-H-capped	-534.65818	-534.44669	-534.43203
f5-12a-H-capped	-555.79213	-555.57170	-555.55788
f13-14a-H-capped	-595.07984	-594.83101	-594.81599
f2-8a-H-capped	-480.63884	-480.42239	-480.40999
f2-8b-H-capped	-688.36708	-688.09511	-688.07657
f12-18a-H-capped	-595.07251	-594.82470	-594.80933

Suppl. Table 1: **G4(MP2)-6X energies of radicals, reference structures and fragments of the PYD crosslink.** The nomenclature follows Suppl. Fig. 1.

Bond	BDE	Rad. (a)	center (a)	RSE (a)	Rad. (b)	center (b)	RSE (b)	RSE sum
f12-18	282.2	f12-18a	C	-58.3	f12-18b	C	-87.1	-145.4
f12-18 depr	220.0	f12-18a depr	C	-101.0	f12-18b	C	-87.1	-188.1
f16-17	306.3	f16-17a	C	-22.2	f16-17b	C	-87.1	-109.3
f14-15	312.5	f14-15a	C	-18.0	f14-15b	C	-87.1	-105.1
f13-14	344.0	f13-14a	C	-43.9	f13-14b	C	-13.2	-57.1
f9-11	353.8	f9-11a	C	-47.4	f9-11b	C	-21.0	-68.4
f8-9	369.7	f8-9a	C	-25.9	f8-9b	C	-46.1	-72.0
f11-16	370.5	f11-16a	C	-15.5	f11-16b	C	-13.2	-28.7
f2-8	436.5	f2-8a	N	13.7	f2-8b	C	-15.8	-2.1
f6-13	456.1	f6-13a	C	54.4	f6-13b	C	-21.0	33.4
f5-12	480.3	f5-12a	C	56.9	f5-12b	C	-13.2	43.7

Suppl. Table 2: **BDEs, RSEs, and summed RSEs of PYD bonds and radicals.** The nomenclature follows Suppl. Fig. 1. All values are given in kJ/mol. BDEs were calculated from enthalpies at 298 K (H298K).

1.2 Data for DPD crosslink

The absent hydroxyl group in DPD did not affect the C_α-C_β bond of the top right arm, implying the same for the C_α-C_β bond of the top left arm. The C_α-C_β bond of the lower arm is closer to the hydroxyl group, but the effect on bonds in γ position should be on the order of the methodological error. Hence, we do not differentiate between low BDEs of PYD and DPD in the manuscript.

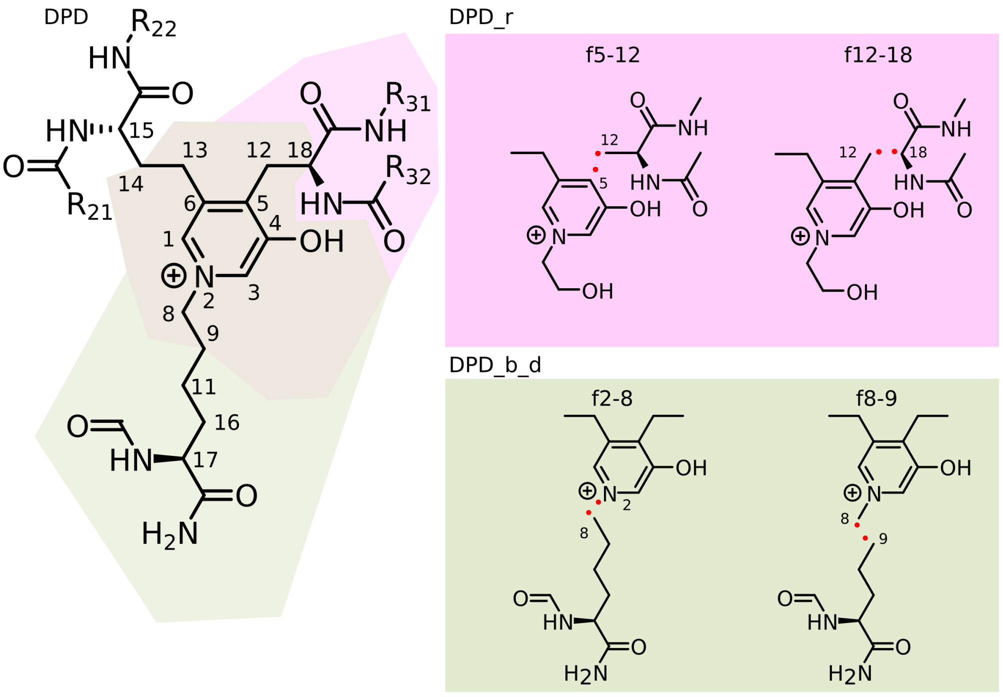

Suppl. Fig. 2: **Internal nomenclature of molecular structures used for calculations of the DPD crosslink.** The presented nomenclature corresponds to the following Suppl. Tables 3 and 4. The ”a” fragment always includes the central nitrogen, the ”b” fragment always the truncated backbone.

Molecule	ZPVE [Ha]	H0K [Ha]	H298K [Ha]
1_DPD_b_d	-1014.10090	-1013.69091	-1013.66534
2_DPD_r	-974.83586	-974.45517	-974.43024
f2-8b	-533.98242	-533.78462	-533.77025
f8-9b	-494.71165	-494.54175	-494.52878
f5-12a	-479.93279	-479.73017	-479.71751
f12-18a	-519.25667	-519.02679	-519.01303
f2-8a	-479.94870	-479.74752	-479.73474
f8-9a	-519.23885	-519.00942	-518.99564
f11-16b	-494.71218	-494.54312	-494.52969
f16-17b	-455.46470	-455.32179	-455.30981

Suppl. Table 3: **G4(MP2)-6X energies of radicals, reference structures and fragments of the DPD crosslink.** The nomenclature follows Suppl. Fig. 2.

Bond	BDE	Rad. (a)	center (a)	Rad. (b)	center (b)
f2-8	-421.0	f2-8a	N	f2-8b	C
f8-9	-370.0	f8-9a	C	f8-9b	C
f5-12	-480.6	f5-12a	C	f5-12b	C
f12-18	-282.0	f12-18a	C	f12-18b	C

Suppl. Table 4: **BDEs of DPD bonds.** The nomenclature follows Suppl. Fig. 2. All values are given in kJ/mol. BDEs were calculated from enthalpies at 298 K (H298K).

1.3 Data for HLKNL crosslink

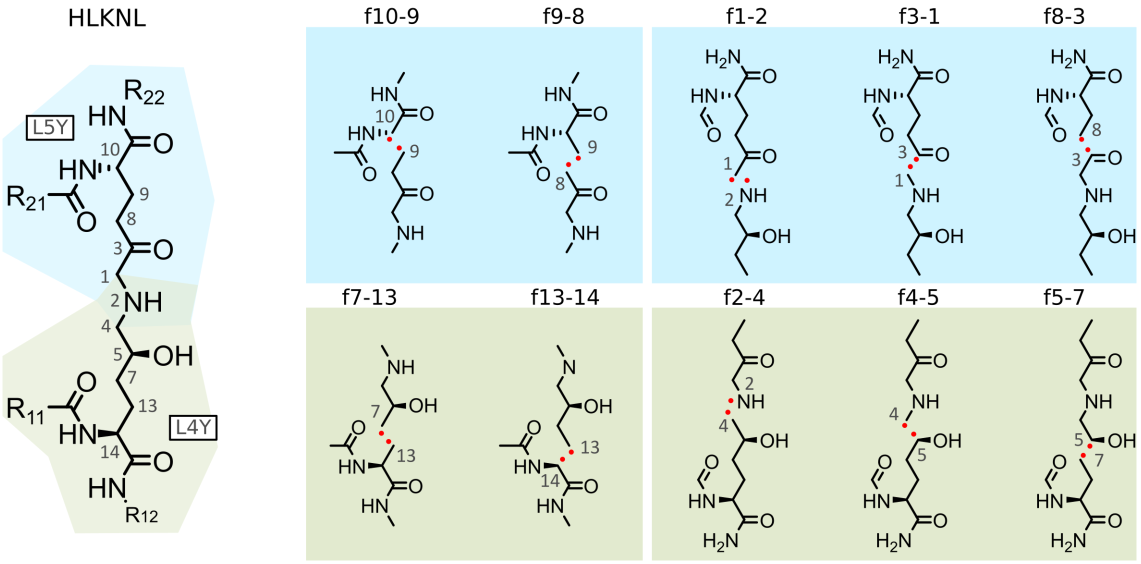

Suppl. Fig. 3: **Internal nomenclature of molecular structures used for BDE calculations of the HLKNL crosslink.** The presented nomenclature corresponds to the Suppl. Tables 5 and 6. The "a" fragment always includes the central nitrogen, the "b" fragment always the truncated backbone.

Molecule	ZPVE [Ha]	H0K [Ha]	H298K [Ha]
p_hlknl_t_long_prod	-896.14927	-895.82246	-895.79977
p_hlknl_b_long_prod	-896.15996	-895.83177	-895.80962
p_hlknl_t_short_prod	-781.71295	-781.41905	-781.39826
p_hlknl_b_short_prod	-782.92794	-782.61089	-782.58965
f8-9b	-569.88602	-569.70896	-569.69538
f1-3a	-327.35260	-327.18660	-327.17616
f1-3b	-568.67824	-568.52396	-568.51081
fd9-11b	-455.44184	-455.29971	-455.28825
f1-2a	-288.07841	-287.94104	-287.93204
f2-8b	-609.14869	-608.94587	-608.93055
f4-5a	-326.13899	-325.99668	-325.98663
f5-7a	-440.57469	-440.39845	-440.38636
f2-4a	-286.86604	-286.75235	-286.74373
f1-2b	-607.94753	-607.76695	-607.75251
f3-8a	-440.57027	-440.39406	-440.38162
f10-9a	-326.12088	-325.97975	-325.96937
f8-9a	-286.86199	-286.74703	-286.73867
f13-14a	-327.33663	-327.17178	-327.16119
f7-13a	-288.06311	-287.92611	-287.91678
f11-16b	-494.71218	-494.54312	-494.52969
f16-17b	-455.46470	-455.32179	-455.30981
f16-17b-H-capped	-456.11102	-455.95560	-455.94338
f2-4b-H-capped	-609.82541	-609.60794	-609.59294
f5-7b-H-capped	-456.11584	-455.95882	-455.94769
f5-7a-H-capped	-441.23646	-441.04690	-441.03479
f11-16b-H-capped	-495.38826	-495.20462	-495.19139
f10-9a-H-capped	-326.79839	-326.64264	-326.63267
f2-4a-H-capped	-287.53826	-287.40996	-287.40128
f13-14a-H-capped	-328.00952	-327.83033	-327.81987
f1-2a-H-capped	-288.75171	-288.59974	-288.59068
f4-5a-H-capped	-326.79839	-326.64264	-326.63267
f3-8a-H-capped	-441.22368	-441.03554	-441.02313
f8-9a-H-capped	-287.52586	-287.39808	-287.38935
f1-3b-H-capped	-569.33021	-569.16414	-569.15097
f4-5b-H-capped	-570.54905	-570.35865	-570.34509
f1-3a-H-capped	-328.00658	-327.82769	-327.81712
f7-13a-H-capped	-288.73962	-288.58827	-288.57920
f1-2b-H-capped	-608.61124	-608.41755	-608.40282

Suppl. Table 5: **G4(MP2)-6X energies of radicals, reference structures and fragments of HLKNL crosslink.** The nomenclature follows Suppl. Fig. 3.

Bond	BDE	Rad. (a)	center (a)	RSE (a)	Rad. (b)	center (b)	RSE (b)	RSE sum
f1-3	296.1	f1-3a	C	-67.7	f1-3b	C	-69.8	-137.5
f1-2	302.5	f1-2a	N	-29.9	f1-2b	C	-43.2	-73.1
f13-14	311.5	f13-14a	C	-21.2	f13-14b	C	-87.1	-108.3
f10-9	312.6	f10-9a	C	-9.1	f10-9b	C	-87.1	-96.2
f4-5	335.0	f4-5a	C	-54.4	f4-5b	C	-44.7	-99.1
f8-9	341.0	f8-9a	C	-42.2	f8-9b	C	-13.2	-55.4
f3-8	341.1	f3-8a	C	-66.2	f3-8b	C	-19.2	-85.4
f5-7	354.5	f5-7a	C	-48.1	f5-7b	C	-19.2	-67.3
f2-4	355.3	f2-4a	N	-32.8	f2-4b	C	-11.4	-44.2
f7-13	375.9	f7-13a	C	-11.4	f7-13b	C	-13.2	-24.6

Suppl. Table 6: **BDEs, RSEs, and summed RSEs of HLKNL bonds and radicals.** The nomenclature follows Suppl. Fig. 3. All values are given in kJ/mol. BDEs were calculated from enthalpies at 298 K (H298K).

1.4 Data for deH-LNL bonds

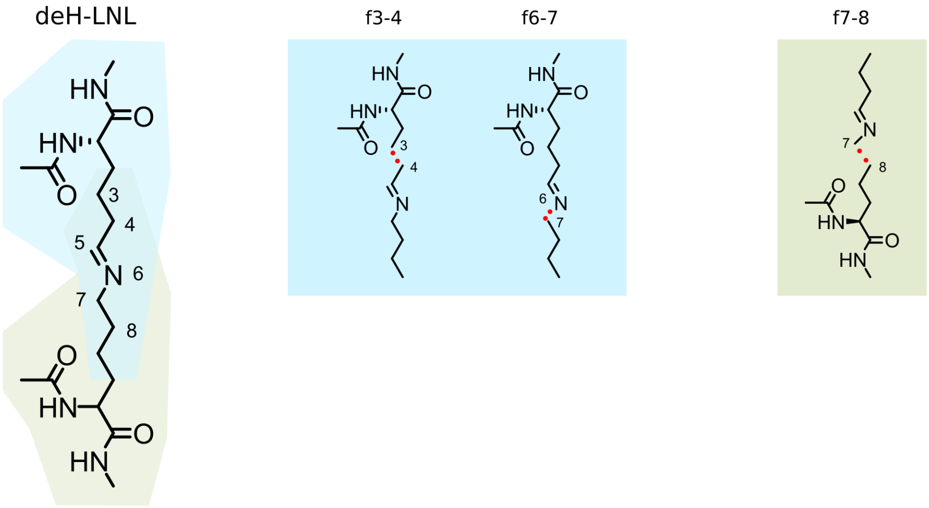

Suppl. Fig. 4: **Internal nomenclature of molecular structures used for BDE calculations of the deH-LNL crosslink.** The presented nomenclature corresponds to the Suppl. Tables 7 and 8. The "a" fragment always includes the central nitrogen, the "b" fragment always the truncated backbone.

Molecule	ZPVE [Ha]	H0K [Ha]	H298K [Ha]
p_deHLNL_LX5_long_prod	-745.80936	-745.49182	-745.47156
p_deHLNL_LX4_long_prod	-745.80937	-745.49200	-745.47170
f6-7a-LX4	-211.70237	-211.59250	-211.58504
f6-7b-LX4	-533.98211	-533.78406	-533.76966
f7-8a	-250.96554	-250.82925	-250.82035
f6-7a-LX5	-588.07866	-587.88532	-587.87073
f7-8b	-494.71125	-494.54110	-494.52803
f6-7b-LX5	-157.60522	-157.49077	-157.48347
f3-4a	-290.23638	-290.07138	-290.06134
f3-4b	-455.44184	-455.29971	-455.28825
f6-7b-H-capped	-534.65632	-534.44364	-534.42971
f3-4a-H-capped	-290.89432	-290.71626	-290.70591
f3-4b-H-capped	-456.11584	-455.95882	-455.94769
f6-7a-H-capped	-212.35615	-212.23261	-212.22513
f7-8a-H-capped	-251.62137	-251.47094	-251.46197
f7-8b-H-capped	-495.38605	-495.20116	-495.18864

Suppl. Table 7: **G4(MP2)-6X energies of radicals, reference structures and fragments of the deH-LNL crosslink.** The nomenclature follows Suppl. Fig. 4.

Bond	BDE	Rad. (a)	center (a)	RSE (a)	Rad. (b)	center (b)	RSE (b)	RSE sum
f6-7	307.2	f6-7a	N	-78.6	f6-7b	C	-17.6	-96.2
f3-4	320.2	f3-4a	C	-58.2	f3-4b	C	-19.2	-77.4
f7-8	323.8	f7-8a	C	-66.0	f7-8b	C	-16.1	-82.1

Suppl. Table 8: **BDEs, RSEs, and summed RSEs of deH-LNL bonds and radicals.** The nomenclature follows Suppl. Fig. 4. All values are given in kJ/mol. BDEs were calculated from enthalpies at 298 K (H298K).

1.5 Data for peptide bonds

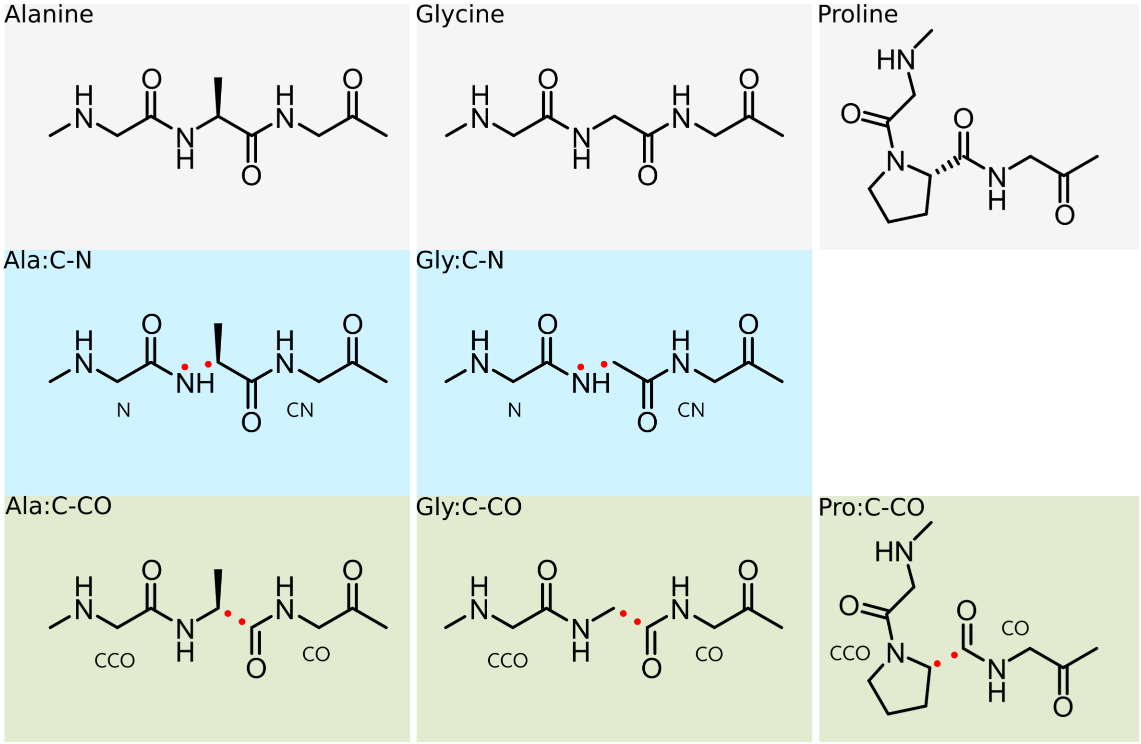

Suppl. Fig. 5: **Internal nomenclature of molecular structures used for BDE calculations of bonds in peptides.** The presented nomenclature corresponds to the Suppl. Tables 9 and 10. Bond scission of a C_α-C bond forms the radicals CCO and CO. Bond scission of the C_α-N bond forms the radicals CN and N.

Molecule	ZPVE [Ha]	H0K [Ha]	H298K [Ha]
ala	-742.44664	-742.18115	-742.16201
gly	-703.17138	-702.93345	-702.91566
pro	-819.76896	-819.46783	-819.44788
ala_CN	-439.39792	-439.24643	-439.23415
gly_CN	-400.12015	-399.99618	-399.98560
pro_CCO	-458.78656	-458.59055	-458.57872
ala_CCO	-381.46144	-381.30155	-381.29019
gly_N	-302.89658	-302.79296	-302.78475
gly_CCO	-342.18689	-342.05462	-342.04485
gly_CO	-360.84817	-360.74991	-360.74087
ala_CN-H-capped	-440.05913	-439.89394	-439.88176
ala_CCO-H-capped	-382.12269	-381.94879	-381.93768
gly_CO-H-capped	-361.51025	-361.40015	-361.39099
pro_CCO-H-capped	-459.44598	-459.23628	-459.22452
gly_N-H-capped	-303.58727	-303.46907	-303.46067
gly_CCO-H-capped	-342.84974	-342.70389	-342.69386
gly_CN-H-capped	-400.78923	-400.65175	-400.64091

Suppl. Table 9: **G4(MP2)-6X energies of peptide fragments and reference structures.** The nomenclature follows Suppl. Fig. 5.

Bond	BDE	Rad. (1)	center (1)	RSE (1)	Rad. (2)	center (2)	RSE (2)	RSE sum
pro_C-CO	336.8	pro_CCO	C	-55.0	pro_CO	C	-43.6	-98.6
gly_C-CO	341.2	gly_CCO	C	-46.6	gly_CO	C	-43.6	-90.2
ala_C-CO	343.8	ala_CCO	C	-50.6	ala_CO	C	-43.6	-94.2
ala_C-N	375.8	ala_CN	C	-50.2	ala_N	N	15.5	-34.7
gly_C-N	381.5	gly_CN	C	-30.0	gly_N	N	15.5	-14.5

Suppl. Table 10: **BDEs, RSEs, and summed RSEs of peptide bonds and radicals.** The nomenclature follows Suppl. Fig. 5. All values are given in kJ/mol. BDEs were calculated from enthalpies at 298 K (H298K).

1.6 Details on breakage site distributions

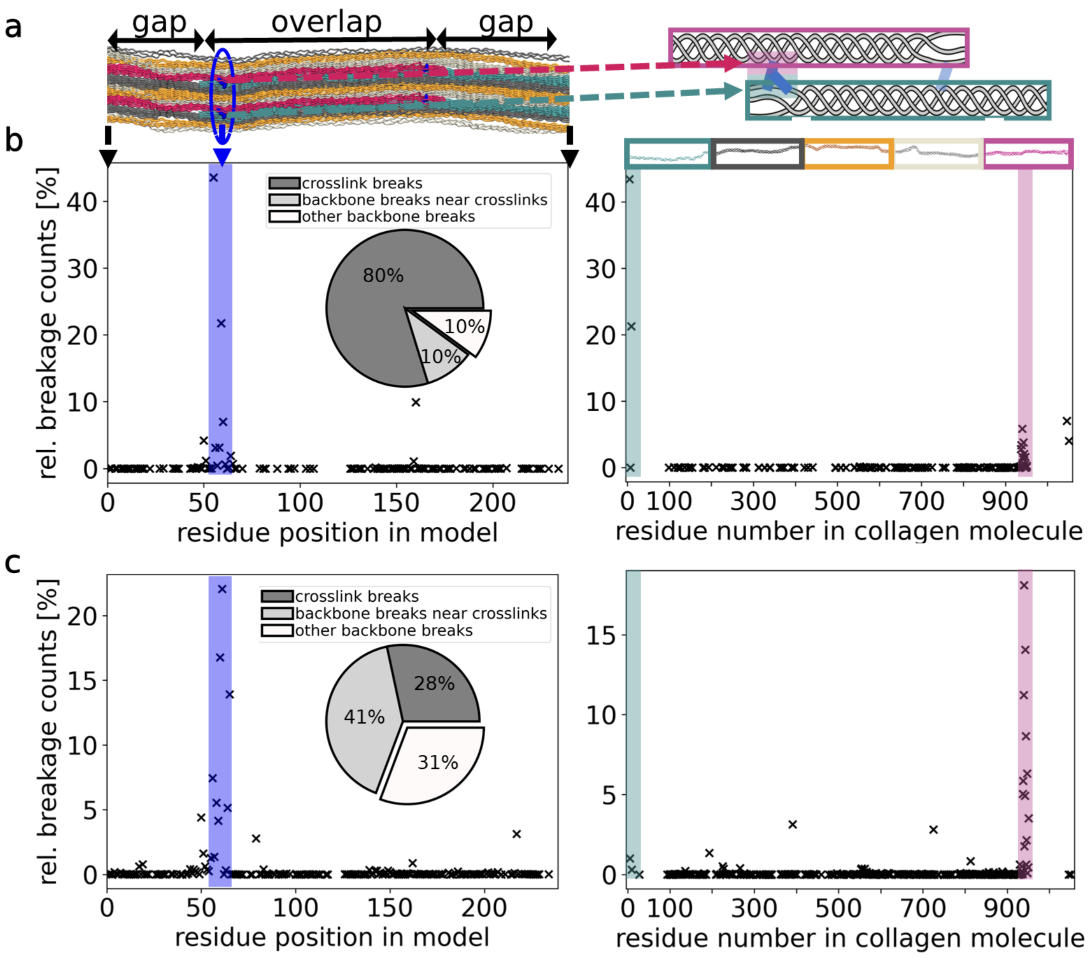

Suppl. Fig. 6: **Comparison of breakage sites in PYD using different BDEs depending on protonation states**. This analysis was performed for data of trivalent (PYD) crosslinks only. **a** Location inside the model (left) and along a collagen triple helix (right) as will be used in b and c. **b** Data for (default) BDE of 220 kJ/mol in the C_α-C_β bond in the shorter arm. N-terminal crosslinks as preferred breakage sites are highlighted in blue in the left panel and, in the right panel, with cyan and magenta depending on the side of the crosslink. **c** Data for BDE of 282 kJ/mol in the C_α-C_β bond in the shorter arm, corresponding to acidic conditions. The ruptures concentrate overall in the same regions, but are more spread out among different residues (also note the different y-axis). In the representation along the collagen molecule on the right, it can be seen how the breakages shift from one of the two arms to the leg of the trivalent crosslink.

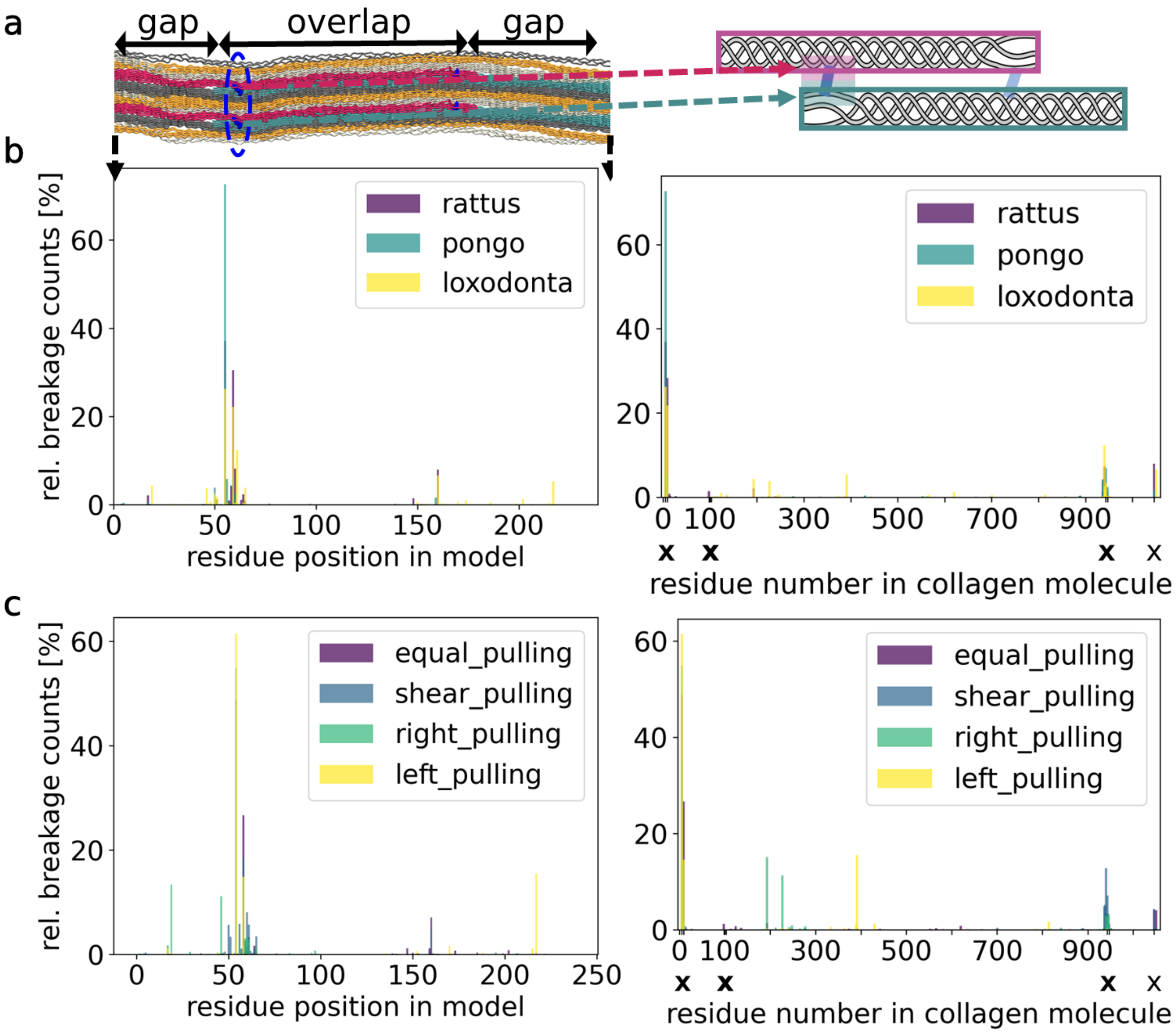

Suppl. Fig. 7: **Comparison of breakage sites depending on model species and pulling setup**. This analysis was performed for the full data set, percentages are normalized within each subset. Crosslink positions are marked with an X in the right panels. **a** Location inside the model (left) and along a collagen triple helix (right) as will be used in b and c. **b** Data for the different model species. The subsets consist of 23 simulations for Rattus, 21 for Pongo and 19 for Loxodonta. **c** Data with respect to the pulling setup. The subsets consists of 6 simulations with divalent crosslinks for each left and right pulling, 28 and 23 simulations with mixed crosslinks for equal and shear pulling, respectively.

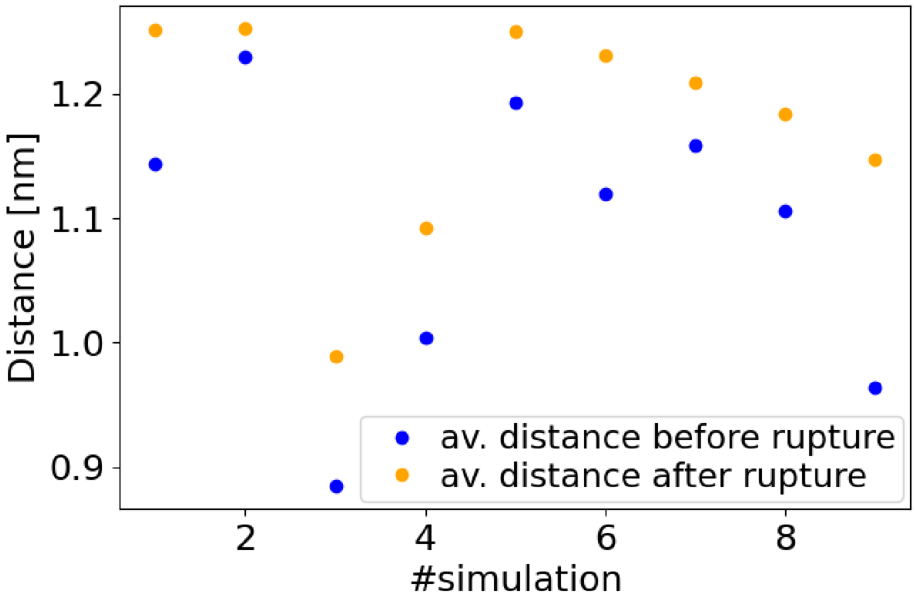

Suppl. Fig. 8: **PYD crosslinks extend after rupture of the short arm.** We measure the distance of C_α atoms of the (not broken) second arm to the C_α atoms of the leg of the PYD crosslink before and after rupturing the shorter first arm. In all replica, the distance increases indicating that the crosslink can now extend by the previously hidden length.

1.7 Details on experiments

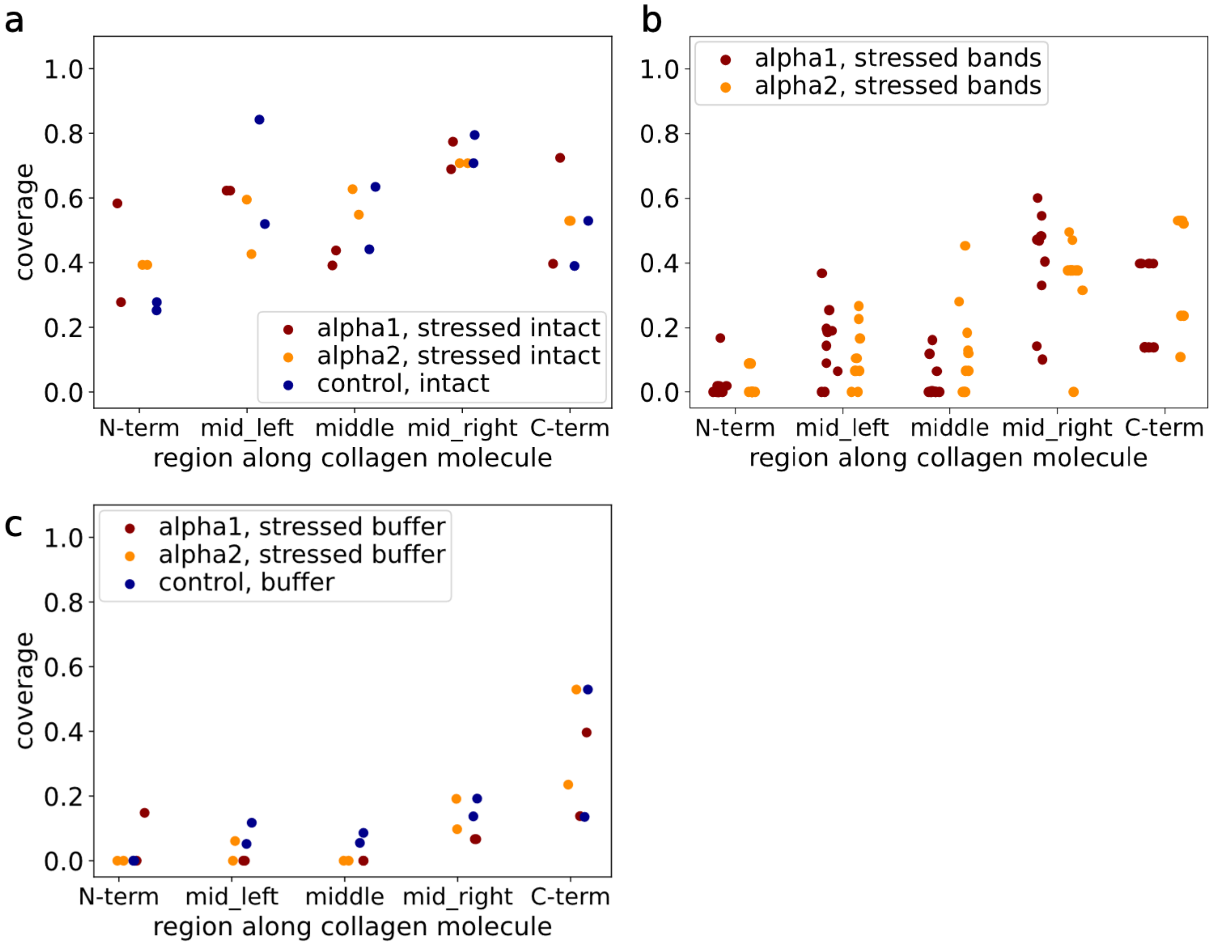

Suppl. Fig. 9: **We find collagen peptides in various areas in our SDS-PAGE gels.** Using mass spectrometry for the different bands, we find collagen fragments of both $\alpha1$(I)- and $\alpha2$(I)-chains from different parts along the collagen sequence. To quantify the coverage, we divided the collagen sequence in 5 regions: Before and after the N-and C-terminal crosslinks (about 100 residues each) and 3 middle parts (about 270 residues each). We conducted this analysis for two different samples each. **a** Sequence coverage for the intact alpha-chains, both in stressed and control, for each sample. **b** Sequence coverage of the 5 identified bands in the stressed samples for each sample. **c** Sequence coverage in the dye front, both control and stressed samples for each sample.

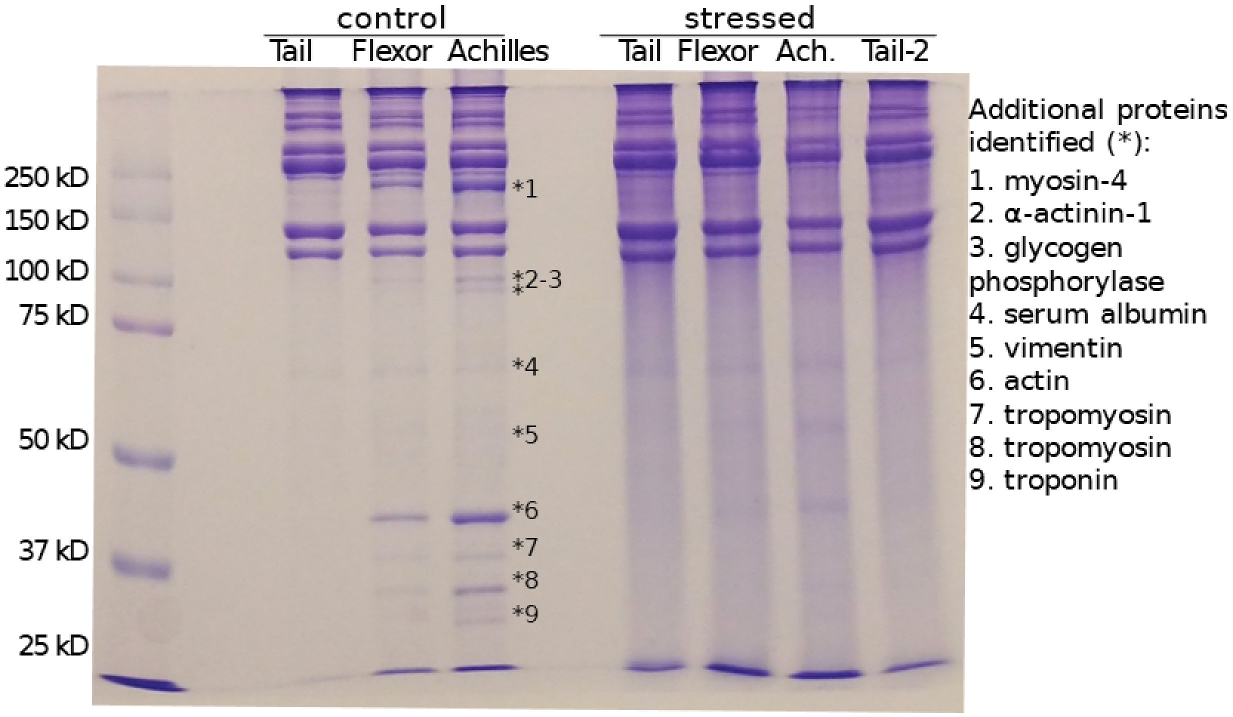

Suppl. Fig. 10: **SDS-PAGE gels of second sample set with different tissue types.** As described in the methods, we compare control and cryo-milled tail, flexor and achilles rat tendon. As biological sample was limited, no further repeats were carried out. In addition to the mass spectrometry analysis for the first gel, we cut out bands deviating from the expected collagen pattern (marked with an asterisk) and find them to be other (muscle) proteins. The intensity analysis of the lanes is part of Fig. 5 in the main article. The uncropped picture is provided in the source data as well.

2 Supplementary Methods

2.1 Software and abbreviations

**Structure design and QM visualizations**	GaussView 5.0.9 [1]
**QM calculations**	Gaussian 09, revision D.01 [2]
**MD simulations**	GROMACS, versions 2018 and 2020 [3]
**Bond rupture simulations**	KIMMDY [4]
**Visualization of molecular structures**	VMD [5]
**Data analysis**	Python 3.8.10, Seaborn 0.11.2 [6]

**PYD**	Pyridinoline
**DPD**	Deoxypyridinoline
**HLKNL**	Hydroxylysino-keto-norleucine
**deH-LNL**	Dehydro-lysino-norleucine
**DOPA**	Dihydroxy-phenylalanines
**BDE**	Bond dissociation energy
**RSE**	Radical stabilization energy
**QM**	Quantum mechanics or quantum mechanical
**DFT**	Density functional theory
**BMK**	Boese-Martin for Kinetics
**MD**	Molecular dynamics
**KIMMDY**	Hybrid Kinetic Monte Carlo/Molecular Dynamics Simulations
**MS**	Mass spectrometry
**EPR**	Electron paramagnetic resonance

2.2 Structure design and electronic structure calculations

2.2.1 Design of molecular structures

Molecular structures for QM calculations were built in GuassView as shown in Suppl. Fig. 1, 2, 3, 4, and 5. By manually deleting atoms beyond broken bonds, pre-optimized radical structures were obtained from optimized molecular structures. H-capped radicals, used for radical stabilization energy calculations, were obtained by hydrogen addition to pre-optimized radical structures. In the Gaussian 09 input files, spin multiplicities were set to 1 for closed shell systems and 2 for radicals.

Naming conventions for collagen crosslinks and crosslink residues were adopted from Obarska-Kosinska et al. [7] with the exception that we extended the Greek letter notation of side-chain carbons into the aromatic system (C_α-C_β-C_γ-C_σ-C_ϵ-C_ζ).

We designed the pre-optimized peptide structures such that the dihedral angles were set to common values of the respective amino acids in a Ramachandran plot of stretched fibrillar collagen type I (Rattus Norvegicus, PYD crosslink at position 9C-5B-944B (N-terminal) and 1046C-1046A-103C (C-terminal)). Initial psi and phi values were $-180°$, $-120°$ for alanine, $-180°$, $-180°$ for glycine, and $-125°$, $-90°$ for proline, respectively. The geometry of pre-optimized PYD and DPD structures was set to geometries found on the ColBuilder website [7]. In that sense, the para alkyl-substituents of the central pyridine moiety were set to be on the same side of the aromatic plane.

2.2.2 Geometry optimization and single point energy calculation (G4(MP2)-6X)

We used a slightly adapted G4(MP2)-6X protocol [8] for all geometry optimizations and energy calculations. The adaptations were the following:

1. Throughout the protocol, we used finer numerical integration grids than proposed in the G4(MP2)-6X example Gaussian input shown in the supplementary file of [8]. We did so because of Bootsma et al.'s [9] findings that Gaussian 09's default numerical integration grid violates the assumption of rotational invariance.

2. The geometry optimization of the G4(MP2)-6X example input file was extended to ensure true geometry convergence and test wavefunction stability. The first geometry optimization often appeared to have converged, but more accurate force constants from the following frequency calculation showed that convergence had not yet been achieved.

3. Other than the use of finer numerical integration grids, adaptations of the energy calculation steps include calculating T1 diagnostics during CCSD(T) steps and not calculating Raman frequencies during the BMK frequency calculation.

Before energy calculation, each electronic structure was checked for convergence in all parameters and to be free of imaginary frequencies. Structures which failed to converge in their geometry were re-optimized with tighter convergence criteria and smaller optimization steps until convergeence under the initial criteria.
The final energies were calculated as described in the original paper with the provided Perl script after non-invasive modifications accounting for our input file format.
Large molecular structures (e.g., PYD substructures) had to be calculated in a two-step process due to time restraints on available computing clusters. In these cases, CCSD(T) calculations were carried out on local workstations over several days or weeks. MP2 and the following calculations were again performed on high-performance computing clusters. Consecutive log files of these calculations were prepared by concatenating both log files. Memory allocations were set dynamically.

2.3 Details of energy calculations

Bond dissociation energies (BDEs) were calculated as described in Equation 1 based on G4(MP2)-6X enthalpies (ΔH_{298K}) of a given molecule (R_1-R_2) and its fragments formed after homolytic bond scission (R_1^*, R_2^*). Energies of radical fragments shared by multiple structures were calculated once and reused in applicable BDE calculations. Examples are identical C_α^*-C_β radicals in the PYD residues LYX, LY3, and LY2.

$$\text{BDE} = \Delta H_{298\text{K}}(\text{R}_1^*) + \Delta H_{298\text{K}}(\text{R}_2^*) - \Delta H_{298\text{K}}(\text{R}_1\text{-R}_2) \tag{1}$$

In this study, radical stabilization energies (RSEs) are formally defined relative to BDEs of CH_3-H bonds for C-centred and NH_2-H bonds for N-centred radicals. G4(MP2)-6X enthalpies of radicals ($\Delta H_{298K}(R_1^*)$, $\Delta H_{298K}(Ref^*)$) and their H-capped counterparts ($\Delta H_{298K}(R_1\text{-}H)$, $\Delta H_{298K}(Ref\text{-}H)$) were used to obtain RSEs as given in Equation (2).

$$\begin{aligned} \text{RSE}(\text{R}_1^*) &= \text{BDE}(\text{R}_1\text{-}H) - \text{BDE}(\text{Ref-}H) \\ &= \Delta H_{298\text{K}}(\text{R}_1^*) - \Delta H_{298\text{K}}(\text{R}_1\text{-}H) - \Delta H_{298\text{K}}(\text{Ref}^*) + \Delta H_{298\text{K}}(\text{Ref-}H) \end{aligned} \tag{2}$$

Used reference energies were also calculated with the G4(MP2-6X) protocol:

1. $\Delta H_{298K}(CH_3^*)$: -39.76049 Ha
2. $\Delta H_{298K}(CH_4)$: -40.42724 Ha
3. $\Delta H_{298K}(NH_2^*)$: -55.80836 Ha
4. $\Delta H_{298K}(NH_3)$: -56.47839 Ha
5. $\Delta H_{298K}(H^*)$: -0.50002 Ha

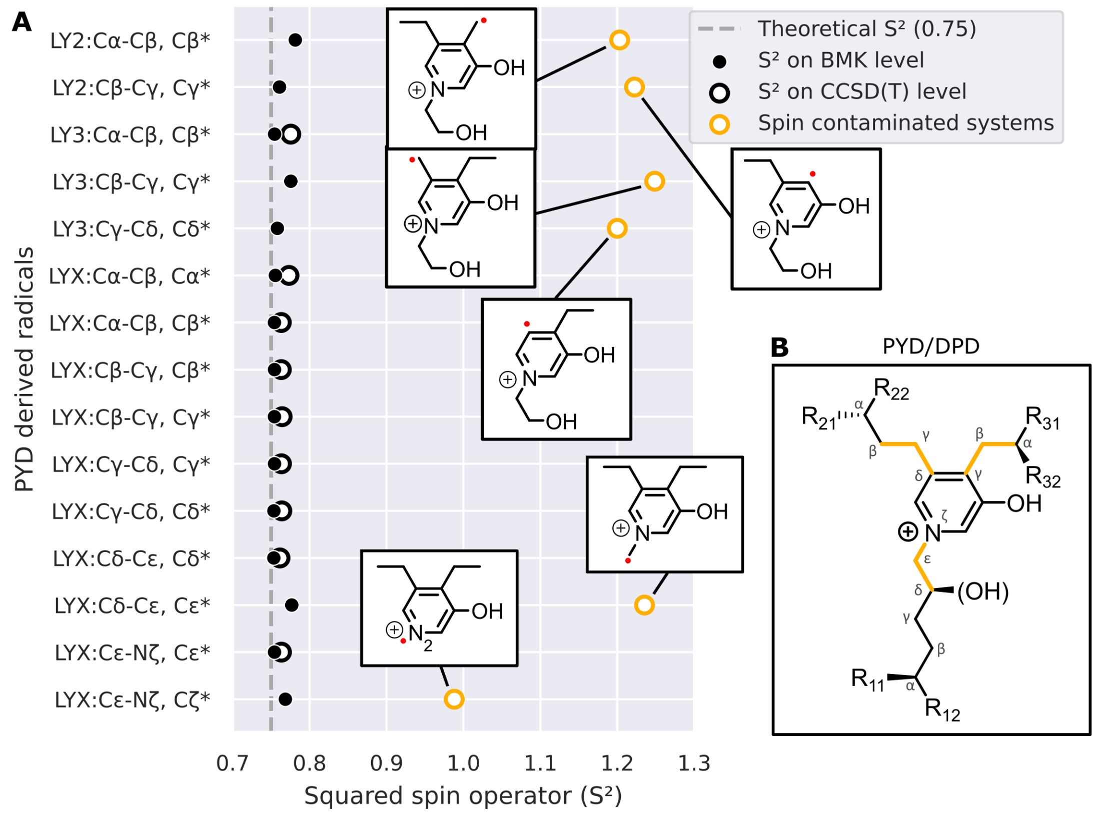

Suppl. Fig. 11: **Some electronic structures are spin contaminated in wave function-based theories.** Values of the squared spin operator (S^2) were automatically calculated during every G4(MP2)-6X protocol step. All shown S^2 values are before the annihilation of the largest spin contaminant. **A** Squared spin operator (S^2) for all unique PYD radicals on the BMK and CCSD(T) level. S^2 values on the CCSD(T) level are representative for all wave function based steps of the G4(MP2)-6X protocol. Highlighted in orange are spin contaminated structures, which all share a proximity of the radical to the aromatic system. Data points shown in black have an acceptable S^2 value, within the limits of 0.75 ± 0.038 [10]. **B** Molecular PYD and DPD structure. BDEs of highlighted bonds (orange) were calculated with a spin contaminated structure and should be carefully considered.

2.4 Discussion of spin contaminated electronic structures

Suppl. Fig. 11A shows the squared spin operator (S^2) values of all PYD radicals, obtained on the DFT (BMK) and wave function (CCSD(T)) level of theory. In nine out of fifteen cases, both wave function and DFT S^2 values are within an acceptable 5 % range [10] of the theoretical value of 0.75. In six cases, however, structures became spin contaminated when transitioning from DFT to wave function theories, as done in the G4(MP2)-6X protocol. For the analysis in this manuscript, two of these six are relevant in the sense that they have BDEs low enough to compete as rupture candidates.

Spin contamination describes the artificial mixing of different spin states in calculated electronic structures, and can be an artifact of unrestricted HF-based wave functions. It can indicate that molecules require multi-reference treatments, that wave functions are unstable [10], and that electronic structures might be inaccurate. Having acceptable S^2 values on the BMK level, but spin contamination of the same structures on the wave function level is not uncommon for DFT functionals with low HF exchange (e.g., BMK) [11]. Interestingly, high S^2 values are exclusively observed for radicals in alpha or beta position to the cationic aromatic system. The radical in alpha position to the positively charged nitrogen shows lower spin contamination

than other spin contaminated structures. Differing S^2 values between BMK and CCSD(T) wave functions indicate that on the BMK level, a doublet spin state was found as the minimal energy state, whereas on the wave function level, it was a mixture with higher-order spin states.
We tested the wave function stability of all contaminated structures on the BMK level, but could not detect any instabilities. For one electronic structure (PYD:LYX:C_σ-C_ϵ^*), we further performed multiple wave function stability tests without finding instabilities. These were based on different checkpoint files of the G4(MP2)-6X protocol (BMK/6-31+G(2df,p) optimization, 2 times BMK/6-31+G(2df,p) frequency calculation, and CCSD(T)/FrzG4 energy calculation) and performed with the keyword "stable" in Gaussian on different levels of theory (BMK/6-31+G(2df,p), HF/6-31+G(2df,p), HF/6-31+G(2df,p), MP2(FrzG4)/GTMP2LargeXP, respectively). Additionally, before and after the BMK to CCSD(T) transition, the system's spin density and charge distribution were visualized but appeared to be virtually identical. T1 diagnostics [12] calculated during the CCSD(T) step were also all within acceptable limits (< 0.045) for open-shell systems [13]. Ideally, we would perform multi-reference calculations to validate or improve contaminated electronic structures. Unfortunately, such calculations are outside the scope of this paper. In their absence, we expect our electronic structures to be acceptable, as optimization and frequency calculations are BMK based and no wave function instabilities or suspicious spin or charge distributions were found. The fact that different spin states were found as the lowest energy states in BMK and wave function-based theories could indicate near degeneracy.

2.5 Strain and convergence in the MD simulations

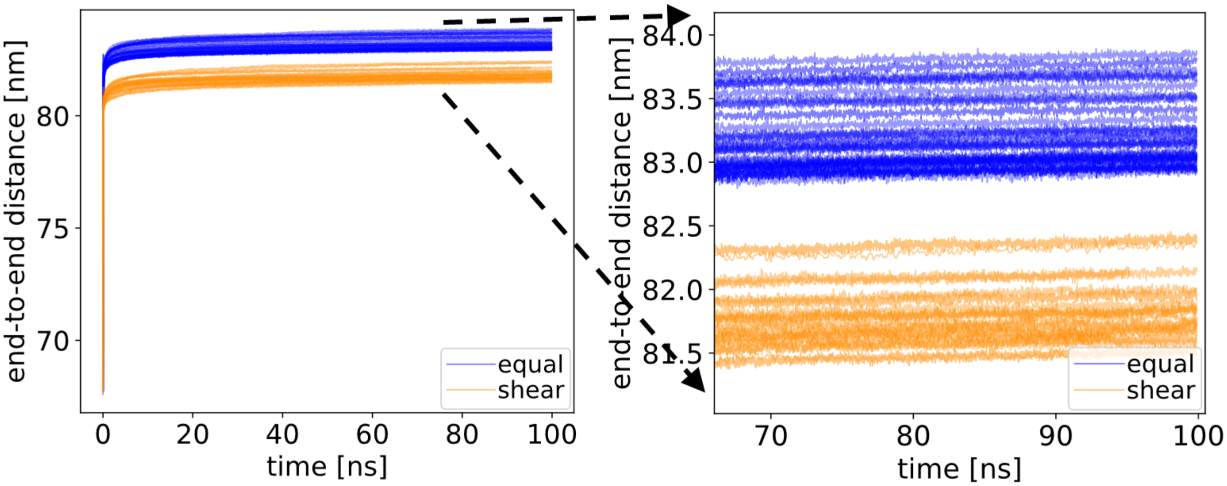

Suppl. Fig. 12: **End-to-End distances of the fibrils in the MD simulations**. Data from setups in which all fibrils are pulled with the same force ('equal') are shown in blue, and data from inhomogeneous pulling setups ('shear', as described in the methods) in orange. On the right side, we zoomed in on the last 30 ns.

In order to test the convergence of the pulling simulations, we monitored the end-to-end distances of the fibrils as shown in Suppl. Fig. 12. While the different batches vary slightly, all fibrils get strongly stretched from the starting length of about 67.5 nm in the beginning and then converge to a certain length — a behavior that is expected due to the immediately applied constant force. From approximately 50 ns onwards, the fibrils show only a very minor further increase relative to the whole fibril length, suggesting that also the internal force distribution has largely converged. Another observation is that the main stretching happens on a time scale in the order of 10 ns, such that microscopically a new stretched (quasi-)equilibrium can be reached quickly. Overall, we reach strains in the range 20%-24%.

2.6 Force calculation in KIMMDY: Force field influence and baseline correction

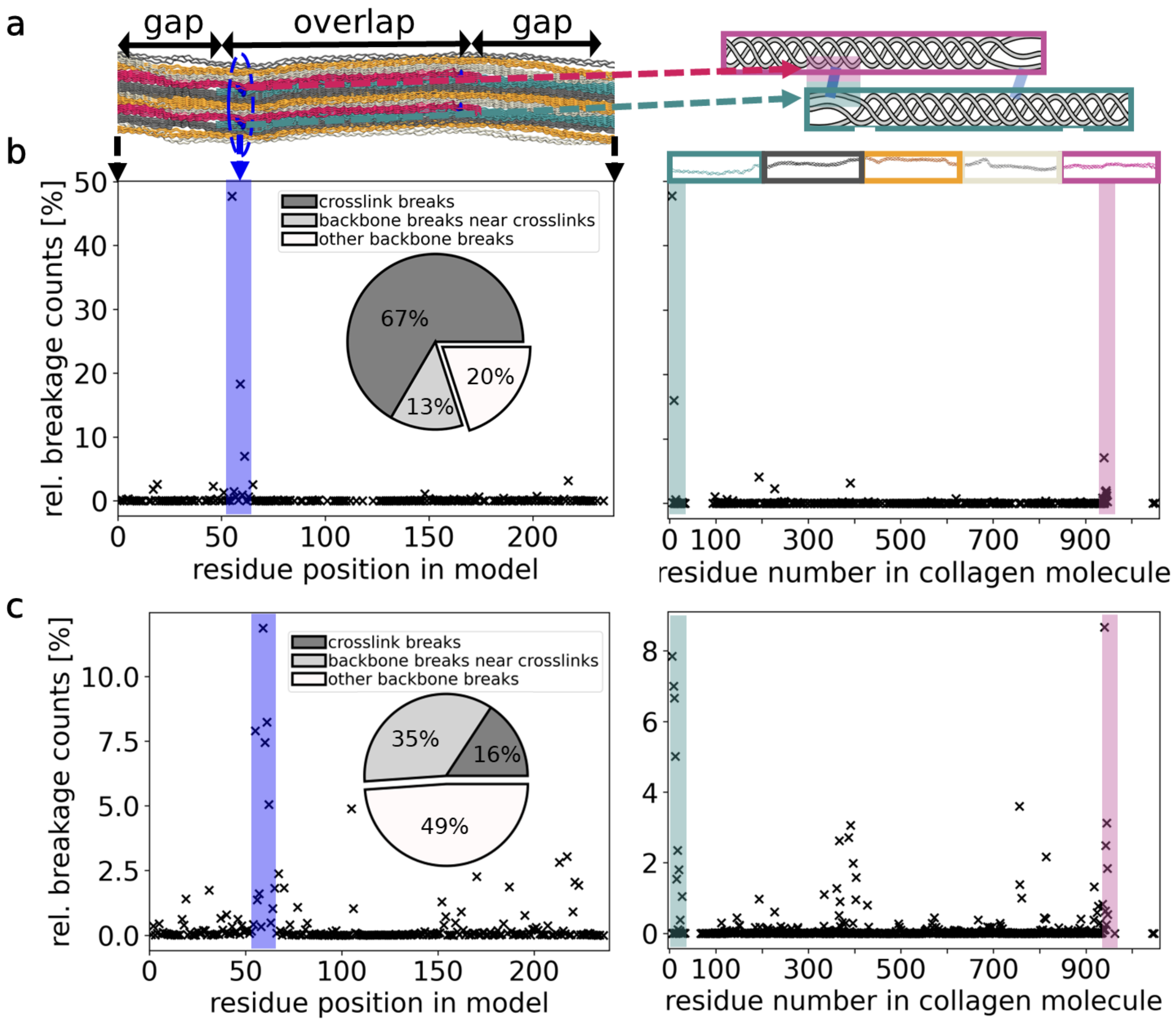

Suppl. Fig. 13: **Comparison of breakage type ratios using different methods to calculate bond forces**. This analysis was performed for data of divalent (HLKNL) crosslinks only. **a** Location inside the model (left) and along a collagen triple helix (right) as will be used in b and c. **b** Data for default force calculation. **c** Data using (maximal) correction factor. The concentration happens at the same regions, but is more spread out among different residues (also note the different y-axis).

As stated in the methods section of the manuscript, we use the bond-breaking simulation scheme KIMMDY [4] and also revised the way it calculates forces during the course of this work. For this, we investigated simple polypeptide chains under force. The naive expectation here is that if pulling from the outside, the measured bond force should be the same in all amino acids inside, if we assume they behave like a chain of hookean springs. However, what we observed is a different picture: Depending on the amino acid type and position, force levels deviated measurably. Even though those differences were not large compared to their absolute levels, their error propagates exponentially in the rate (as rates are exponentially dependent on the effective energy barrier). We suspect two underlying reasons for the deviations: First, there are other bonded force field terms (angle, dihedrals) that are neglected in KIMMDY and are heterogeneous among

amino acids. This was corroborated by the fact that the effect varied when repeating the same calculation with Charmm36 force field [14]. This would mean that it is an artifact that should ideally be corrected for. Second, there are still other interactions ongoing in a simple polypeptide chain and the assumption of a Hookean spring does not fully hold. So, what we measure are actual differences ongoing in the molecules. We also found evidence for this: Depending on the amino acids sequence in the polypeptide, i.e. the neighboring amino acids, the effects changed. This points us to the idea that the side chains might influence the also the backbone bonds, e.g. via interactions with neighbors leading to a stretch towards one direction.

Overall, there is probably a force-field contribution due to the nature of KIMMDY and a real contribution, which hardly can be separated by the nature of the protocol. To estimate an upper bound of the effect, we used the aforementioned polypeptide simulations as baseline reference and added a new force correction into KIMMDY, that calculates the bond forces amino acid type dependent. As this counters the effect fully, this is probably overcorrecting and mainly used for comparison in the following, which is why we provide the respective figures here in the supplement. Whereas there are quantitatively large changes on a small scale (e.g. with the correction, breakages happen in more different bonds next to each other rather in a few amino acid types where the forces used to be higher, compare Suppl. Fig. 13), already on a mid-size scale this effect seems to be small compared against the effect of the actual force distribution. So the results in this manuscript, aiming at the identification of weak regions in collagen, are robust against these considerations. Note that as stated, this is an upper limit only and the actual effect size will be smaller.

2.7 Sequence alignment of collagen types I, III, V in Rattus Norvegicus

Whereas our atomistic models are collagen type I, there are also other collagen types mixed into fibrils, for example type III and V. For Rattus Norvegicus, we have carried out a multiple sequence alignment analysis of these types using Clustal Omega [15]. In Suppl. Fig. 14, we have focused on the terminal regions, as they contain the crosslinks we are mostly interested in here. We find both the lysine-derived crosslinks sites and also the adjacent potential radical scavenging DOPA sites (i.e. Tyr and Phe) highly conserved. This is consistent with previous work on human collagen and on conversation of crosslink sites across species [16, 17] and indicates that our findings might generalize to other collagen types.

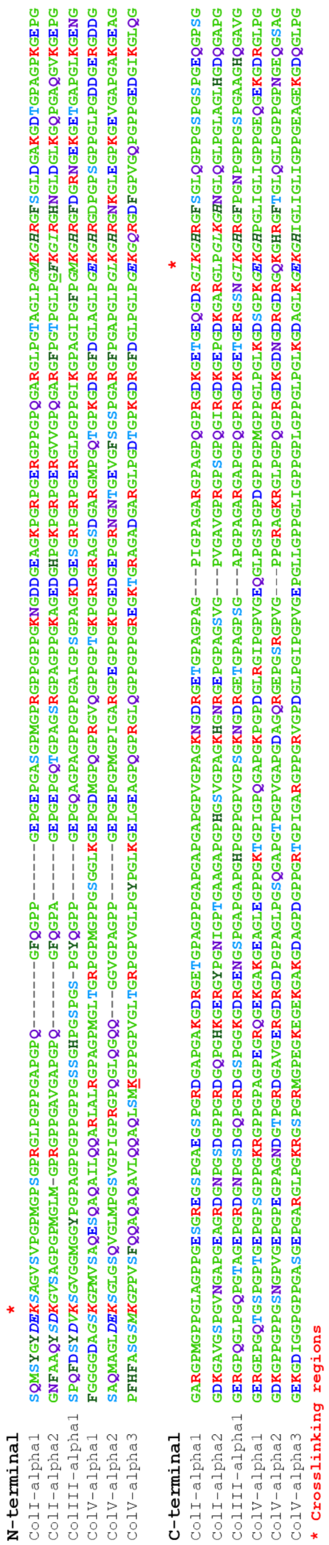

Suppl. Fig. 14: **Sequence alignment of collagen types I, III, V in Rattus Norvegicus**. We show the terminal sites containing the lysine-derived crosslink positions.

References

1. Roy Dennington Todd Keith, J. M. *GaussView* Computer Program. http://semichem.com/aboutus.php. 2009.
2. Frisch, M. J. *et al.* Gaussian 09, revision A. 1. *Gaussian Inc. Wallingford CT* **27,** 34 (2009).
3. Abraham, M. J. *et al.* GROMACS: High performance molecular simulations through multi-level parallelism from laptops to supercomputers. *SoftwareX* **1,** 19–25 (2015).
4. Rennekamp, B., Kutzki, F., Obarska-Kosinska, A., Zapp, C. & Gräter, F. Hybrid Kinetic Monte Carlo/Molecular Dynamics Simulations of Bond Scissions in Proteins. *Journal of Chemical Theory and Computation* **16,** 553–563. ISSN: 1549-9618. `https://dx.doi.org/10.1021/acs.jctc.9b00786%20https://pubs.acs.org/doi/pdf/10.1021/acs.jctc.9b00786` (2020).
5. Humphrey, W., Dalke, A. & Schulten, K. VMD – Visual Molecular Dynamics. *Journal of Molecular Graphics* **14,** 33–38 (1996).
6. Waskom, M. L. seaborn: statistical data visualization. *Journal of Open Source Software* **6,** 3021. `https://doi.org/10.21105/joss.03021` (2021).
7. Obarska-Kosinska, A., Rennekamp, B., Ünal, A. & Grater, F. ColBuilder: a server to build collagen fibril models. *Biophys J* **120,** 3544–3549. ISSN: 1542-0086 (Electronic) 0006-3495 (Linking). `https://www.ncbi.nlm.nih.gov/pubmed/34265261` (2021).
8. Chan, B., Deng, J. & Radom, L. G4(MP2)-6X: A Cost-Effective Improvement to G4(MP2). *Journal of Chemical Theory and Computation* **7,** 112–120. ISSN: 1549-9618. `https://dx.doi.org/10.1021/ct100542x` (2011).
9. Bootsma, A. N. & Wheeler, S. Popular Integration Grids Can Result in Large Errors in DFT-Computed Free Energies. `https://dx.doi.org/10.26434/chemrxiv.8864204.v1%20https://chemrxiv.org/engage/api-gateway/chemrxiv/assets/orp/resource/item/60c7436f0f50dba3a3395ef2/original/popular-integration-grids-can-result-in-large-errors-in-dft-computed-free-energies.pdf` (2019).
10. Cramer, C. J. *Essentials of Computational Chemistry: Theories and Models* 2nd Edition. ISBN: 978-0-470-09182-1 (John Wiley & Sons, Ltd, The Atrium, Southern Gate, Chichester, West Sussex PO198SQ, England, 2004).
11. Baker, J., Scheiner, A. & Andzelm, J. Spin contamination in density functional theory. *Chemical Physics Letters* **216,** 380–388. ISSN: 0009-2614. `https://www.sciencedirect.com/science/article/pii/000926149390113F` (1993).
12. Lee, T. J. & Taylor, P. R. A diagnostic for determining the quality of single-reference electron correlation methods. *International Journal of Quantum Chemistry* **36.** T1 Diagnostic, 199–207. ISSN: 0020-7608. `https://dx.doi.org/10.1002/qua.560360824%20https://onlinelibrary.wiley.com/doi/pdfdirect/10.1002/qua.560360824?download=true` (1998).
13. Rienstra-Kiracofe, J. C., Allen, W. D. & Schaefer, H. F. The C2H5+ O2Reaction Mechanism: High-Level ab Initio Characterizations. *The Journal of Physical Chemistry A* **104.** T1 value for radical systems, 9823–9840. ISSN: 1089-5639. `https://dx.doi.org/10.1021/jp001041k` (2000).
14. Huang, J. *et al.* CHARMM36m: an improved force field for folded and intrinsically disordered proteins. en. *Nat. Methods* **14,** 71–73 (Jan. 2017).
15. Madeira, F. *et al.* Search and sequence analysis tools services from EMBL-EBI in 2022. *Nucleic Acids Research* **50,** W276–W279. `https://doi.org/10.1093/nar/gkac240` (Apr. 2022).
16. Eyre, D. R. & Wu, J.-J. in *Topics in Current Chemistry* 207–229 (Springer Berlin Heidelberg, Apr. 2005). `https://doi.org/10.1007/b103828`.
17. Rodriguez-Pascual, F. & Slatter, D. A. Collagen cross-linking: insights on the evolution of metazoan extracellular matrix. *Scientific Reports* **6.** `https://doi.org/10.1038/srep37374` (Nov. 2016).
