# Supplementary material for: Collagen breaks at weak sacrificial bonds taming its mechanoradicals
Source: Nat Commun. 2023 Apr 12;14:2075. doi: 10.1038/s41467-023-37726-z (PMC10097693; doi:10.1038/s41467-023-37726-z)
Supplement: Supplementary file 3 — Reporting Summary [file 41467_2023_37726_MOESM3_ESM.pdf]

nature portfolio

Corresponding author(s): Frauke Gräter

Last updated by author(s): Mar 24, 2023

Reporting Summary

Nature Portfolio wishes to improve the reproducibility of the work that we publish. This form provides structure for consistency and transparency in reporting. For further information on Nature Portfolio policies, see our Editorial Policies and the Editorial Policy Checklist.

Statistics

For all statistical analyses, confirm that the following items are present in the figure legend, table legend, main text, or Methods section.

n/a	Confirmed	
☐	☒	The exact sample size (*n*) for each experimental group/condition, given as a discrete number and unit of measurement
☐	☒	A statement on whether measurements were taken from distinct samples or whether the same sample was measured repeatedly
☒	☐	The statistical test(s) used AND whether they are one- or two-sided<br>*Only common tests should be described solely by name; describe more complex techniques in the Methods section.*
☒	☐	A description of all covariates tested
☐	☒	A description of any assumptions or corrections, such as tests of normality and adjustment for multiple comparisons
☐	☒	A full description of the statistical parameters including central tendency (e.g. means) or other basic estimates (e.g. regression coefficient) AND variation (e.g. standard deviation) or associated estimates of uncertainty (e.g. confidence intervals)
☒	☐	For null hypothesis testing, the test statistic (e.g. *F*, *t*, *r*) with confidence intervals, effect sizes, degrees of freedom and *P* value noted<br>*Give P values as exact values whenever suitable.*
☒	☐	For Bayesian analysis, information on the choice of priors and Markov chain Monte Carlo settings
☐	☒	For hierarchical and complex designs, identification of the appropriate level for tests and full reporting of outcomes
☒	☐	Estimates of effect sizes (e.g. Cohen's *d*, Pearson's *r*), indicating how they were calculated

Our web collection on statistics for biologists contains articles on many of the points above.

Software and code

Policy information about availability of computer code

Data collection	- MD simulations: GROMACS versions: 2020.3, 2020.5 patched with PLUMED 2.7.2, 2018.6, 2018.1 patched with PLUMED 2.4.2<br>- QM simulations: Gaussian 09, revision D.01<br>- KIMMDY version 1.0 (available on GitHub)<br>- Experimental: Thermo Proteome Discoverer 2.4 0.305, Thermo Xcalibur 4.3.73.11
Data analysis	- Data analysis: Python 3.8.10, Seaborn 0.11.2<br>- Visualization: VMD 1.9.3, GaussView 5.0.9<br>- Gel quantification: ImageJ 1.53t

For manuscripts utilizing custom algorithms or software that are central to the research but not yet described in published literature, software must be made available to editors and reviewers. We strongly encourage code deposition in a community repository (e.g. GitHub). See the Nature Portfolio guidelines for submitting code & software for further information.

Data

Policy information about availability of data

All manuscripts must include a data availability statement. This statement should provide the following information, where applicable:

- Accession codes, unique identifiers, or web links for publicly available datasets
- A description of any restrictions on data availability
- For clinical datasets or third party data, please ensure that the statement adheres to our policy

The QM data generated in this study (including data for Fig. 1) is provided in the supplementary tables. Run input files (enabling reproduction) for MD and KIMMDY simulations used in this study, derived breakage counts per simulation (including data for the Figs. 2-4) and experimental data (for Fig. 5) and uncropped pictures of gels are all available in a heiDATA repository [63]: https://doi.org/10.11588/data/HJ6SVM . Full raw MD simulation data is too large to deposit and available on request. Amino acid sequence for Col1a1 and Col1a2 were obtained from the Ensembl database: https://useast.ensembl.org/Rattus norvegicus/Info/Index

Human research participants

Policy information about studies involving human research participants and Sex and Gender in Research.

Reporting on sex and gender	n.a.
Population characteristics	n.a.
Recruitment	n.a.
Ethics oversight	n.a.

Note that full information on the approval of the study protocol must also be provided in the manuscript.

Field-specific reporting

Please select the one below that is the best fit for your research. If you are not sure, read the appropriate sections before making your selection.

☒ Life sciences ☐ Behavioural & social sciences ☐ Ecological, evolutionary & environmental sciences

For a reference copy of the document with all sections, see nature.com/documents/nr-reporting-summary-flat.pdf

Life sciences study design

All studies must disclose on these points even when the disclosure is negative.

Sample size	Biological material from several rats was prepared as much as feasible/available, and pooled for shipment for both the first and second set of experimental data. From the first set, 4 technical replicates were taken for the first SDS page gels. From the second set, another 4 lanes per control and treatment group were conducted. No sample size calculation was performed. All replica, both of the same and different tissues, showed the same trend so that the sample size was sufficient.
Data exclusions	No data was excluded.
Replication	Same trends were observed in all 4 technical replicates of the first sample set as well as between the 3 tissue types in the second sample set. Beside that, no further replication was conducted.
Randomization	Biological material was pooled to ensure random mixture of samples from different animals.
Blinding	Blinding was not conducted. Sample uptake in the treated cases varied, so this would not have been possible.

Reporting for specific materials, systems and methods

We require information from authors about some types of materials, experimental systems and methods used in many studies. Here, indicate whether each material, system or method listed is relevant to your study. If you are not sure if a list item applies to your research, read the appropriate section before selecting a response.

Materials & experimental systems

n/a	Involved in the study
☒	☐ Antibodies
☒	☐ Eukaryotic cell lines
☒	☐ Palaeontology and archaeology
☐	☒ Animals and other organisms
☒	☐ Clinical data
☒	☐ Dual use research of concern

Methods

n/a	Involved in the study
☒	☐ ChIP-seq
☒	☐ Flow cytometry
☒	☐ MRI-based neuroimaging

Animals and other research organisms

Policy information about studies involving animals; ARRIVE guidelines recommended for reporting animal research, and Sex and Gender in Research

Laboratory animals	Rat (Rattus Norvegicus and Wister)
Wild animals	No wild animals involved.
Reporting on sex	Only female rats were used to avoid breeding, but findings should generalize.
Field-collected samples	No field-collected samples
Ethics oversight	No ethics approval was needed, as the tendons were donated.

Note that full information on the approval of the study protocol must also be provided in the manuscript.
